# Integrating depression management into HIV primary care in central Malawi: the implementation of a pilot capacity building program

**DOI:** 10.1186/s12913-018-3388-z

**Published:** 2018-07-31

**Authors:** Michael Udedi, Melissa A. Stockton, Kazione Kulisewa, Mina C. Hosseinipour, Bradley N. Gaynes, Steven M. Mphonda, Beatrice Matanje Mwagomba, Alick C. Mazenga, Brian W. Pence

**Affiliations:** 1 NCDs & Mental Health Unit, Ministry of Health, Ministry of Health, P. O. Box 30377 Capital City, Lilongwe 3, Malawi; 2 Epidemiology Department, University of North Carolina at Chapel Hill Gillings School of Global Public Health, 135 Dauer Dr, Chapel Hill, NC 27599 USA; 3 Ministry of Health, Kamuzu Central Hospital and Bwaila Psychiatric Hospital, Private Bag, 149 Lilongwe, Malawi; 4 University of North Carolina Project-Malawi, Tidziwe Centre, Private Bag, A-104 Lilongwe, Malawi; 5 Department of Psychiatry, University of North Carolina at Chapel Hill School of Medicine, 333 S Columbia St, Chapel Hill, NC 27516 USA; 6 Lighthouse Trust, Kamuzu Central Hospital, P.O. Box 106, Lilongwe, Malawi; 7 Baylor College of Medicine Abbott Fund Children’s Clinical Centre of Excellence, Kamuzu Central Hospital, Private Bag, B-397 Lilongwe, Malawi

**Keywords:** HIV/AIDS, Mental health, Depression, Sub-Saharan Africa, Malawi, Integration, Service delivery, Implementation science

## Abstract

**Background:**

In Malawi, early retention in HIV care remains challenging. Depression is strongly associated with reduced anti-retroviral therapy (ART) adherence and viral suppression. Appropriate depression care for people initiating ART is likely to be supportive of early and continued engagement in the HIV care continuum. This paper aims to provide an overview of a task-shifting program that integrates depression screening and treatment into HIV care and the strategy used to evaluate this program, describes the implementation process, and discusses key challenges and lessons learned in the first phase of program implementation.

**Methods:**

We are implementing a program integrating depression screening and treatment into HIV care initiation at two clinics in Lilongwe District, Malawi. The program’s effect on patients’ depression and HIV outcomes will be evaluated using a multiple baseline pre-post study. In this manuscript, we draw from our experiences as program implementers and some of the quantitative data to describe the process of implementation and key lessons learned.

**Results:**

We successfully implemented the screening phase of this program at both clinics; 88.3 and 93.2% of newly diagnosed patients have been screened for depression at each clinic respectively. 25% of enrolled patients reported symptoms of mild-to-severe depression and only 6% reported symptoms of moderate-to-severe depression. Key lessons learned from the process show the importance of utilizing existing processes and infrastructure and focusing on iterative and collaborative learning. We continued to face challenges around establishing a sense of program ownership among providers, developing capacity to diagnose and manage depression, and ensuring the availability of appropriate medication. Our efforts to address these challenges provide insight into the technical and managerial support needed to prepare for, roll out, and sustain integrated models of mental health and HIV care.

**Conclusions:**

This activity demonstrates how a depression screening program can successfully be integrated into HIV care within the public health system in Malawi. While this program focuses on integrating depression management into HIV care, most of the lessons learned could apply to integration of mental health into any non-psychiatric specialist setting.

**Trial registration:**

ClinicalTrials.gov ID [NCT03555669]. Retrospectively registered on 13 June 2018.

**Electronic supplementary material:**

The online version of this article (10.1186/s12913-018-3388-z) contains supplementary material, which is available to authorized users.

## Background

In Malawi a growing body of research is beginning to document the scope of mental health challenges in the general Malawian population. In Malawian primary care settings, 28.8% of all patients (regardless of human immunodeficiency virus (HIV) status) have a common mental disorder [1]; the most common condition appears to be depression. Prevalence estimates of depression among populations living with HIV vary: 19% among adolescents attending HIV clinics [2]; 16% among newly initiating ART adults; and 9% among adults on anti-retroviral therapy (ART) for at least 6 months [3]. However, the lack of national data documenting the magnitude of mental health problems has historically hampered efforts to secure and allocate resources for mental health services. Mental health care infrastructure and human resources in Malawi are limited; there are only four mental health facilities in the country (two public and two private), − only three of which are currently operational – all located in urban centers. Mental health care is treated as a specialized service in Malawi and is primarily offered from these aforementioned facilities.

In response to the public health burden of mental health disorders in Malawi, Malawi’s Health Sector Strategic Plan (HSSP 2011–2016) formally recognized the need to address non-communicable diseases (NCDs) in Malawi [4] and established the NCDs and Mental Health Unit of the Ministry of Health (MOH) in the 2011–12 financial year. Further, the 2017–2022 Malawi Health Sector Strategic Plan prioritizes treatment for mental health, including depression treatment, under their Essential Health Package [5, 6]. The NCDs and Mental Health Unit of the MOH developed action plans to 1) integrate mental health services into other general health services; 2) improve the capacity of general health care workers through training to diagnose and manage mental health conditions at different levels of care; and 3) raise awareness of mental health disorders and treatment among the general population through community health workers, teachers, religious leaders, peer educators and the media [7]. As a result of these efforts, a pilot program aimed at building the mental health capacity of community health workers, encouraging community-level mental health promotion and detection, and integrating these community health worker activities into the primary care setting was successfully implemented in Zomba, Malawi [8–10]. However, further efforts are needed to effectively realize the MOH’s mental health goals.

Simultaneously, early retention in HIV care is a major obstacle to achieving the Joint United Nations Programme on HIV/AIDS (UNAIDS) 90–90-90 goals [11–13]. The first year of ART is a particularly vulnerable period: nearly a quarter of people initiating ART are lost to care within the first 12 months [11–13]. Among people initiating ART, those with comorbid depression are a large and especially vulnerable population. Depression affects 18 to 30% of patients receiving HIV care in Africa [14] and is an important barrier to early ART retention [15, 16]. Depression is also strongly associated with reduced ART adherence and viral suppression [14, 17–20] as well as greater perceived stigma and faster HIV clinical progression [17, 20–23] and suicide [24–26]. While evidence of the impact of depression treatment on HIV care retention and treatment outcomes in Africa is currently lacking, a burgeoning body of research suggests appropriate depression care for people initiating ART is likely to be important for early and continued engagement in the HIV care continuum [15, 27–29].

There has been a recent push to integrate mental health services into existing primary and community health services to address the mental health burden among people living with HIV in resource limited settings [30–32]. In sub-Saharan Africa, where research on integrating services has been limited, task-shifting approaches are an effective, cost-effective, and practical means of managing depression [30, 33]. In Zimbabwe and South Africa, training lay health workers to provide psychosocial therapy to people living with HIV was effective at reducing symptoms of depression [34, 35]. In Cameroon and Tanzania, training HIV care providers to prescribe and manage antidepressant treatment proved effective at improving depression outcomes [36–38]. These findings suggest that integrating depression screening and treatment in HIV care services in sub-Saharan Africa is both feasible and acceptable.

In light of the particular vulnerability to depression of people living with HIV and the Malawian government’s commitment to addressing mental health, the Malawi MOH proposed integrating depression screening and treatment into HIV care using a task-shifting model. The MOH designed a pilot treatment program in partnership with the University of North Carolina (UNC) at Chapel Hill, UNC Project-Malawi, and Lighthouse Trust with the generous support of the United States President’s Emergency Plan for AIDS Relief (PEPFAR) through the United States Agency for International Development’s (USAID) Supporting Operational AIDS Research (Project SOAR). The treatment program combines two resource-efficient, context-appropriate depression treatment models — algorithm-based care for depression (ABCD) medication management and problem-solving therapy (PST) — into a single, stepped-care program that will be offered to patients newly initiating antiretroviral treatment (ART) and who have depressive symptoms. While a few studies from the sub-Saharan region have proven the feasibility, acceptability, and effectiveness of integrating depression management into HIV care [34–38], there is a dearth of practical guidance on how to design, implement and evaluate such programs. To fill this knowledge gap, this paper provides an overview of the methods used to design Malawi’s pilot program including a description of the program itself, the site selection, the evaluation strategy, and ethical considerations. We then describe the resulting implementation process and key challenges and lessons learned in the first phase of program implementation drawing both from our own experiences and some of the initial quantitative data collected.

## Methods

### Program overview, site selection, evaluation design

#### Program overview

We designed the program to be implemented in two phases – a screening phase and an intervention phase. Throughout the entirety of the program, all adult patients who present for HIV testing and counseling (HTC) and test positive for HIV are screened for depression. The program excluded pregnant women who test for HIV at the maternity ward as part of antenatal care.

At the beginning of the screening phase, the program team held an in-person, lecture-style training to teach providers to screen patients for depression and re-oriented providers to the standard care options for managing depressed patients, namely, referral for counseling and prescription of antidepressants (amitriptyline). As well, the team taught providers how to recognize, triage, and respond to individuals with suicidal ideation. Specifically, providers were taught how to distinguish between passive and active suicidal thoughts, how to assess level of risk (e.g. specific plans, means) and protective factors (e.g. social support), and about available treatment and referral options.

During the intervention phase, the team will train providers to provide ABCD and community health workers will be trained to provide PST. ABCD is a resource-efficient, task-shifting model for delivering high quality, effective, safe antidepressant management in non-psychiatric settings using standardize metrics. ABCD has been effectively incorporated into HIV care in high-income countries [39], and more recently in Tanzania [38], Uganda [40], and Cameroon [37]. These prior efforts among HIV-positive patients in Sub-Saharan Africa have relied on tricyclic antidepressants such as amitriptyline [37, 38] and imipramine [40] which have demonstrated excellent safety and efficacy profiles in these populations. PST is a psychological treatment that teaches patients how to identify triggers and effectively manage stressful life events by learning or reactivating problem solving skills [41]. PST has also proven to be a successful depression treatment in the region, as seen in the “Friendship Bench” project with community health workers in Zimbabwe [42].

#### Site selection

The MOH is the main organization responsible for public health care provision in Malawi. Malawi has a three-tier public health care system which is comprised of: four central hospitals; 28 district, community and rural hospitals; and over 700 health centers. At the primary level, health centers serve the majority of the country’s rural and semi urban population. While health centers across the country vary in size and in the range of available services, they often house an HIV clinic, an out-patient department and a maternal and child health department or offer such services. At these health centers, the majority of services are provided by nurses and clinicians who do not specialize in one area, but rotate through every department or by community health workers. Mental health services are centralized and are often provided in specialized tertiary health facilities. However, there is a dearth of specialized psychiatric health providers or health workers adequately trained to treat mental disorders such as depression. To address this shortage of human resources, the MOH is advocating for the integration of mental health services into the primary level. Specifically, the MOH would like to build the capacity of the nurses, clinicians and community health workers to identify and manage mental health disorders as part of regular service provision at public health centers.

The proposed depression screening and treatment program is currently being implemented into two HIV clinics – Area 18 and Area 25 – in Lilongwe, Malawi. The two clinics were chosen by the MOH in liaison with the Lilongwe District Health Office (DHO) which manages the health services in the district. (Table 1) These clinics are nested within two semi-urban primary level health centers under Lilongwe District Health Management, in the Central Region of Malawi. Area 25 health center serves a catchment population of 63, 783 adults and children while Area 18 health center has a catchment population of 212,160 adults and children. However, anyone can receive health services at either facilities free of charge, regardless of where they live. Both sites use electronic medical record (EMR) systems and host non-governmental partners that provide technical supervision and support, similar to what is seen at other public health facilities across the country. The team chose these sites because they are considered by the MOH to be representative of primary level health centers in the Malawian health system and since they serve a mixed urban, semi urban and rural population. Additionally, the availability of EMR data will simplify the evaluation and these facilities are equipped to host such a program.

**Table 1:** Clinic Characteristics

	Area 18	Area 25
HTC and ART Staff	≈44	≈62
New ART patient registered from July 2016–June 2017	1194	1768
Adult ART Clinic	Tuesdays & Thursdays	
Permanent Psychiatric Services	Psychiatric nurse on staff	none
Outreach Psychiatric services	Mission clinic health held monthly	
Available Medication	Amitriptyline stocked irregularly	Chronic supply shortage

*HTC* HIV testing and counseling, *ART* Anti-retroviral therapy

#### Evaluation design

**Fig. 1:**
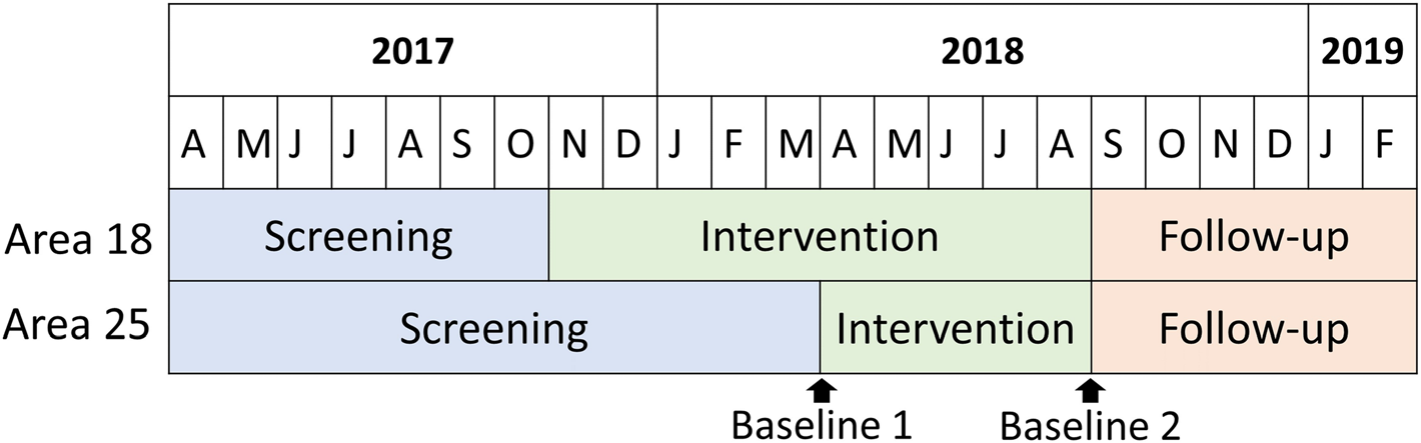
Evaluation Design

The program’s impact on HIV and depression outcomes will be evaluated using a multiple baseline pre-post study. In each clinic, screening for depression began prior to launching the intervention phase in order to accrue a comparison group. Patients who screen positive for depression during the “screening” period will comprise the comparison group, while patients who screen positive during the “intervention” period will comprise the active treatment group. However, the intervention will then be rolled out at different time points at the two clinics. Thus, the baseline “screening” period for will be longer at one clinic than for the other. (Fig. 1). We chose a multiple baseline design as it can provide evidence of causal relationships in settings where a randomized controlled trial is not feasible. The multiple baseline design is also stronger than a simple pre-post design because it provides stronger control for temporal trends while also allowing each clinic to serve as its own control.

In total, we plan to enroll 2300 new ART initiators. Evaluation team will abstract clinical data on depression treatment, depression outcomes, attendance of HIV appointments, HIV treatment, and viral loads for all consenting newly diagnosed patients from their clinical records. This data will be cleaned and analyzed using appropriate statistical software. The effect of depression treatment on mental health and HIV outcomes will be analyzed using methods appropriate for a dichotomous outcome and a multiple baseline design. Ultimately, we will compare mental health and HIV outcomes before (among the screening group) and after (among the intervention group) the integration of the depression treatment program.

In this manuscript, we will draw on our experiences as program implementers to describe the process of implementation and key lessons learned, and will also include some of the initial findings from the quantitative data collected.

#### Ethical considerations

The program team sought and obtained ethical approval from both the Malawi MOH’s National Health Science Research Committee (NHSRC) institutional review board (IRB) and the UNC IRB.

## Results

**Fig. 2:**
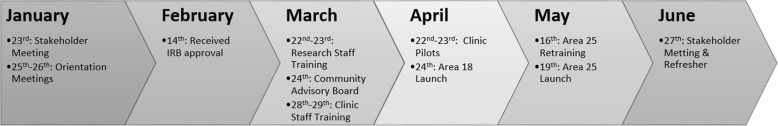
Implementation Timeline

In this section we describe the implementation process including the original stakeholder meeting and site visits, instrument adaptation, clinic trainings, and the implementation itself (Fig. 2).

### Implementation process

#### Stakeholder meeting

The program team hosted a mental health roundtable discussion on January 23rd, 2017, to mobilize key stakeholders involved in the provision of HIV and mental health. The program implementation staff, representatives from the MOH, UNC-Chapel Hill, UNC Project-Malawi, the University of Malawi College of Medicine, and Lighthouse Trust, and management staff from Area 18 and Area 25 clinics attended the discussion. Over the course of the meeting, the MOH officials provided an overview of their mental health priorities and attendees presented their ongoing and developing projects relating to mental health in Malawi. The roundtable provided an opportunity for the MOH to introduce the depression screening and treatment program, discuss the current state of mental health and HIV care provision, and identify key stakeholders’ concerns and priorities. A summary document produced from this meeting captured these key themes as well as ideas on how to take Malawi’s broader mental health agenda forward. The key stakeholders agreed to come together again in the coming year to continue this discussion in hopes of growing the nidus of investment in mental health care in Malawi.

#### Site visits and clinic sensitization meetings

The implementation team visited both study sites to meet with clinic leadership and provide mental health sensitization trainings to clinic staff on January 25th and 26th 2017. During these site visits, the team gathered information on the clinic infrastructure, staffing of the HTC and ART clinics, staffing of psychiatric nurses, patient flow, medication availability and supply chain. These insights then informed the development of appropriate instruments and protocols.

The principal investigators led two-hour sensitization meetings with available clinic staff. During these meetings, the program team provided an introduction to depression and its prevalence, its intersection with HIV, and how it can be diagnosed and treated. These sensitization meetings also provided the program team an opportunity to introduce the depression treatment program to the clinics early on and elicit staff feedback on depression and its effect on engagement in HIV care in Malawi.

#### Instrument adaptation and protocol development

The Patient Health Questionnaire-9 (PHQ-9) was the instrument selected to screen patients for depression. The PHQ-9 is a widely used nine item instrument that assesses the presence of nine symptoms of depression within the previous two-weeks as specified by the Diagnostic and Statistical Manual of Mental Disorders IV [43]. Each of the nine items are scored from 0 (not at all) to 3 (nearly every day). The first two questions – known as the Patient Health Questionnaire-2 (PHQ-2) – capture depressed mood and anhedonia, the two core symptoms of depression. Only if a patient scores above 0 on the PHQ-2, will that patient be assessed for the presence of the remaining seven symptoms of the PHQ-9. A total score of 5–9, 10–14, 15–20, or 20 and above are considered indicative of mild, moderate, moderately severe, or severe depression, respectively [44]. The PHQ-9 is one of a number of mental health screening tools that have been used in Malawi and other African countries, including the Edinburgh Postnatal Depression Scale (designed for the perinatal period) and the Kessler-10 and Self-Reporting Questionnaire, which screen for common mental disorders.

The PHQ-9 was chosen because it focuses specifically on depression, has been widely used and validated in many different settings [45], and works well both as a screening tool for depression as well as a longitudinal measure to monitor response to treatment. This tool has been validated for use in HIV-positive populations in other countries in the region [46, 47]. While not validated, the PHQ-9 has been used in one other study in Malawi [3].

The team augmented the standard PHQ-9 with an additional screening protocol to probe the degree of severity of suicidality in patients who screened positive for suicidal ideation on the PHQ-9 (Additional file 1). Both the PHQ-9 and associated suicide risk assessment protocol were translated into Chichewa, the vernacular language in the central region of Malawi. This has entailed an iterative process involving the health workers to ensure that the PHQ-9 adequately captures the depressive symptoms yet is culturally understandable. The translated instrument is currently being validated.

The team also developed clinic materials to support implementation. After examining the HIV clinical forms (called mastercards) used to record HIV care, the team developed a mental health clinical form in a similar style (Additional file 2). As well, the team developed clinic reference guide posters to remind providers how to administer and interpret the PHQ-9. These posters hang on the walls of the ART clinic rooms (Additional file 3).

After assessing patient flow and staffing, the program team developed a protocol that delineated staff responsibilities for screening patients and established a process for enrolling patients in the evaluation study. This protocol was presented to clinic leadership and staff and adapted as necessary. The HTC counselors screen all patients receiving HTC services who test positive for HIV with the PHQ-2. Should that patient score above 0 on the PHQ-2, the HTC escorts that patient to a HIV provider to administer the patient with rest of the PHQ-9. At Area 25, the ART clinic is staffed daily, so patients are normally escorted directly to the ART clinic. However, at Area 18, the ART clinic is only staffed with providers on clinic days. Thus, the HTC counselors are charged with locating an available provider at one of the other clinic wards to administer the rest of the PHQ-9 on Mondays and Fridays. The evaluation staff approach all patients newly diagnosed with HIV regardless of their PHQ-2 score while they are waiting to receive treatment or attend a group counseling session, in an effort not to disrupt clinic flow. The staff informs these patients about the program and invites them to allow their clinical data to be abstracted and used to evaluate the program. The evaluation staff then consents all interested patients in a private location.

#### Clinic trainings

Clinic staff trainings were held on March 28th and 29th 2017. The program team members, the MOH principal investigator and a psychiatrist led the trainings which were attended by HIV care providers, HTC counselors, Lighthouse and MOH staff. Thirty-four participants attended the training at Area 18 and 43 participants attended the training at Area 25, about 75 and 70% of the clinics’ HTC and ART staff respectively.

The trainings provided attendees with an overview of the study, an introduction to depression and other mental health conditions, instructions on how to use the PHQ-9, guidance on triaging and responding to suicidal thoughts, treatment options, use of the Mental Health Mastercard, and an overview of how screening would be integrated into patient flow. The trainings also provided an opportunity for staff to raise concerns with the protocol and voice any anticipated challenges. Since then, one refresher training has been held at each clinic, one in May and one in June.

#### Implementation

The program was piloted at both clinics on April 22nd and 23rd 2017 and then officially launched at Area 18 on April 24th. As of the December 1st, 2017, HTC counselors have screened 88.3 and 93.2% of newly diagnosed patients with the PHQ-2 at Area 18 and Area 25 clinics, respectively. Of those patients who score above 0 on the PHQ-2, Area 18 and Area 25 clinics have screened 91.4 and 88.0% with the entire PHQ-9, respectively. Some initial challenges arose including:

- Managing patient flow on non-clinic days: With test-and-treat, patients who test positive for HIV are taken to the ART clinic to initiative care. If no nurses or clinical officers were at the ART clinic, the clerk would often give the patients ART, even though the provision of clinical care is outside of their prevue. On these occasions, no one at the ART clinic was able to complete the PHQ-9 for those with PHQ-2 scores greater than 0 or address any resulting clinical needs. The program staff raised this challenge with clinic leadership who instructed the HTC counselors to bring patients to providers in the outpatient ward to initiate ART and complete the PHQ-9 screening if necessary. This also eliminated the need for the clerk to provide patients with medication.
- Inconsistent administration of the screening tools: Some providers struggled to administer the PHQ-9 in a standardized manner which lead to inter-rater differences in scoring. For example, the program team observed that providers would rephrase the questions when administering the tool and varied in their interpretation patients responses. The research staff provided guidance and ongoing clinical supervision to improve inter-rater reliability.
- Discomfort treating depression: Providers expressed to the program team that they did not feel comfortable treating the cases of depression they identified. Providers were instructed to manage suspected cases of depression using existing resources at their health centers, or refer to the psychiatric nurse.
- Unfamiliarity with new clinical forms: The program introduced new clinical materials clinicians were either only introduced to during the training or were seeing for the first time on the job. Program staff provided guidance on using the Mental Health Mastercard.
- Inconsistent assessment of patient with suicidal thoughts: Initially, providers were not systematically assessing suicide risk, but were each using their own method to decide whether a patient needed to be referred for more intensive psychiatric treatment. Suicide risk assessment was translated into Chichewa and the program team instructed providers to focus on ascertaining whether the patient is actually thinking of hurting themselves.
- Reluctance to integrate of depression management into HIV care: Traditionally mental health care has been the domain of specialized psychiatric providers and providers are already charged with a heavy workload. As such, some providers perceived depression screening as an additional responsibility, for which they were not being additionally compensated. The program team engaged the clinic staff on the importance of depression management in HIV care and worked with staff to find ways to make the integration as easy as possible.

The team assessed the roll-out and addressed any initial challenges before retraining and launching at Area 25 on May 16th and 19th. As of December 1st, 2017, 1160 patients have enrolled in ART care and 1042 patients (90%) have been screened with the PHQ-2. Of those 1042 patients who were screened, a total of 487 patients at Area 18 and 513 at Area 25 consented to have their clinical data used to evaluate the program. At ART initiation, 25% of patients had a PHQ-9 score of 5 or above, indicating mild-to-severe depression. (Table 2).

**Table 2:** Enrollment Indicators

	Area 18 (*N* = 487)				Area 25 (*N* = 513)			
PHQ-9 Score	0	1–4^a^	5-9^b^	≥ 10^c^	0	1–4^1^	5-9^b^	≥ 10^c^
Total (%)	232(48)	133(27)	95(20)	27(6)	280(55)	103(20)	99(19)	31(6)

^a^depressed mood and or anhedonia; ^b^Mild depression; ^c^Moderate-to-severe Depression

### Lesson learned: Successes

#### Formative investigation of existing processes and infrastructure

##### Attention to clinic infrastructure, patient flow and staff responsibilities

Prior to developing a protocol, the program team carefully documented the process patients underwent when testing and initiating HIV care as well as the existing infrastructure at both clinics. After establishing where and when patients are seen by the HTC counselors, the group counselors, the ART registration staff, and providers, the team identified the appropriate staff to screen patients with the PHQ-2 and administer the PHQ-9. They also identified the most convenient times to consent patients for the evaluation component without extending the amount of time they spend at the clinics. In this manner, prior to launching the program, the team laid groundwork to successfully implement without interrupting patient flow or overburdening staff and only minor tweaks to address the availability of clinicians and nurses were needed over the first couple weeks of implementation.

#### Iterative and collaborative learning

##### Cultivating an iterative and collaborative learning environment

To inform clinic leadership and staff about progress, elicit their concerns, and discuss any challenges, the program team holds weekly meetings alternatingly at both clinics. As well, the team holds a weekly operations call with the program team members from the MOH, UNC at Chapel Hill, UNC-Project, and Lighthouse, with other key-stakeholders invited from Baylor College of Medicine. Further highlighting the importance of collaboration, it became evident from the early weeks of implementation at Area 18 that the clinics were staffed not only by MOH employed public health providers, but by rotating staff that report to non-governmental organizations such as Baylor and Lighthouse. In order to assure continuity of program knowledge, all of the rotating staff needed to be trained on the protocol. In response, the program team trained Baylor staff in early September, is working to coordinate orientation training for Lighthouse staff, and communicates weekly with Baylor and Lighthouse leadership.

### Lessons learned: Challenges

#### Fostering ownership

##### Developing clinical ownership of the program

One of the key challenges the team faced during implementation has been clinic resistance to additional responsibilities without additional compensation or incentives. This is unsurprising, as the most visible staff are the evaluators employed by UNC-Project Malawi, an organization that has been involved in public health research in the region for over three decades. In response, the team has continuously emphasized to the clinic staff that the project is a government initiative and has had MOH representation at clinic sensitization meetings, orientation trainings, refreshers and many of the weekly clinic meetings. As well, the team engaged with the Lilongwe DHO and now a District Health Management Team (DHMT) representative is a permanent member of the program team. To supplement this effort, the program team identified supportive clinic staff who demonstrated an ability to support their colleagues implement the screening initiative and nominated these individuals to become on-the-ground mental health champions. These individuals will champion the implementation of the program in the clinics. Ultimately, the extent to which clinic staff and leadership accept this program as government initiative and part of their responsibility as government employees will determine its success.

#### Ongoing capacity building

##### Training clinical staff

Providing quality screening and treatment for depression to patients initiating HIV care requires mental health-literate staff capable of using the screening instruments and following treatment protocols. Thus, to implement this program, all of the clinic staff involved in providing HIV care need to be trained on the protocol, on their responsibilities in the depression screening and treatment process, and on how to use screening tools. In fact, one of the main benefits of this program is the mental health capacity building accomplished through this type of continued education. However, ensuring that all staff were available to attend the training was particularly challenging. At both clinics, providers rotate through the clinic wards, serving some weeks in the out-patient or maternity wards, and others in the HIV clinic, and so forth. As well, the trainings took place while the clinic was open and operating, so many providers were unable to leave their posts to attend. The limited and over-burdened human resources at each clinic further exacerbated this challenge. To ensure that all staff were appropriately trained, the program team held multiple trainings, offered one-on-one orientations, held a refresher training two-months following implementation, and have planned future refresher trainings. To reinforce these efforts, a psychiatrist is scheduled to shadow both the HTC counselors and the providers while they administer the PHQ-2 and PHQ-9. This will provide continued opportunities for one-on-one feedback and training.

#### Mobilization of human resources and medication supplies

##### Provision of specialized psychiatric services

Implementing this program has also highlighted the importance of maintaining a functioning referral system for accessible psychiatric care and ensuring the availability of psychiatric medications. To ensure that patients are able to access and receive appropriate care for depression, the program wanted to ensure that psychiatric care was offered at each clinic at least once a week. This was not a challenge at Area 18, where a psychiatric nurse is scheduled at least once a week and rotates through the ART clinic. However, at Area 25, psychiatric care is only offered by a private outreach team monthly or bi-monthly. Since this challenge was identified during the orientation trainings in March, the program team has been working with the district health management team members to ensure a general mental health clinic is offered weekly.

##### Medication availability

The mental health roundtable discussion in January 2017 raised issues around the availability of medication to treat depression at both the clinic and district level in Malawi during and at the time of the initial site visits, neither of the clinics had stocked amitriptyline or fluoxetine. Both of these drugs should be available as part of Malawi’s Essential Health Package. The program team began working to address challenge immediately, months before launching the program. The program team communicated the supply chain concerns to the clinic leadership, who both agreed to request amitriptyline from the Lilongwe DHO. Communication with the clinic leadership continued, but by early April, neither of the clinics had received a supply of amitriptyline. After discussions with the pharmacy-in-charges at both clinics and the Lilongwe DHO, it became clear the Lilongwe DHO pharmacy had depleted their own supply of amitriptyline. The program’s MOH principal investigator liaised with the Malawi Central Medical Stores Trust on the situation and learned they, too, had depleted their stocks, but they promised to float an emergency psychotropic tender. In light of the nationwide shortage, the clinics requested that the program obtain a starter pack of amitriptyline while waiting normal supplies. Thus, the program bought two 1000 25 mg tablet bottles for each clinic, approximately a 30-day supply for about 30 patients (depending on dosage) per clinic. The MOH is continuing to work with the DHO to ensure that amitriptyline is appropriately stocked.

#### Effectively utilizing existing processes and clinical tools

##### Identifying and reassessing depressed patients

The program integrated depression management into the paper clinical records system, but not into the EMR system. Providers struggled to identify patients with depression and ensure they were reassessed with the PHQ-9 when they returned for ART care each month. When ART patients return to receive care, providers are meant to both update the patient’s EMR and their paper ART Mastercard. However, providers do not necessarily pull the patient’s ART Mastercard – and thus the Mental Health Mastercard – opting instead to use the EMR system, which does not prompt providers to reassess depression. Anticipating this challenge, a green sticker is placed on the health passport of every patient that scores 5 or above on the PHQ-9 to help remind providers which patients are in need of depression care. However, it became clear that returning patients were being missed. Only some providers were aware that they should look for the green sticker. To address this issue early on, the program team raised this challenge at the weekly clinic meetings and asked clinic staff to brainstorm solutions. As a result, the clinic staff decided they need a refresher training in June 2017, data clerks should be instructed to look out for the green stickers and try to help pull the appropriate files, and requested the program team post reminders in the clinic rooms explaining the meaning of the green sticker. As well, providers empower patients to call attention to the green sticker on their health passports when they return to collect their ART medication. As this issue has remained challenging, signs highlighting the last four digits of the ART IDs of depressed patients expected to return each week are also posted in the ART clinic each week by evaluation staff, though this solution is not sustainable. As well, the program team is investigating whether a new module for the EMR system can be developed in recognition that the more this program can be integrated into existing systems and routine clinical responsibilities, the easier it will be for providers to implement.

#### Conducting implementation science research in a clinical setting

##### Separation of clinical and evaluation responsibilities

A key feature of this program is that, while the evaluation staff works on the ground to consent patients and collect data, none of the evaluation staff are meant to have any clinical responsibilities or influence the provision of HIV or mental health care. As the clinics are short staffed and the clinic providers are overburdened, particularly at Area 18, the providers lean on the evaluation team to help patients navigate accessing appropriate care. For example, if an HTC provider is busy and the ART clinic is not staffed (such as on Mondays and Fridays), they will ask an evaluation staff member to help a patient who scored above 0 on the PHQ-2 find an available nurse or clinician in the out-patient or maternity wards. As well, on the pediatric ART clinic days, ART providers may ask newly initiating adults that need to be screened with the PHQ-9 to wait until the end of the day or to return the following day to receive care unless an evaluation staff member can find an available provider. It is particularly important for the sake of sustainability that the evaluation team does not create systems or provide support that would cease to exist at the end of the program. This distinction may not be well appreciated by the clinic staff or leadership, who frequently suggest during the weekly clinic meetings that the evaluation team support their clinical responsibilities. In order to address this issue, the program team highlights the separation of responsibilities in all of the trainings and weekly clinic meetings and MOH investigator, visiting psychiatrist and DHMT representative continue to reinforce this message.

##### Ethical standards for implementation science research

As mentioned ethical approval was sought and obtained from the Malawi MOH’s NHSRC IRB. However, the program team proposed that the program evaluation be considered an implementation science study. As implementation science is concerned with methods to promote the adoption and integration of evidence-based practices into routine health care [48], the team requested to waive the need for individual consent, arguing the evaluation was not human research. The team specifically contended that the program is designed to evaluate the provision of “standard depression care,” not an experimental treatment, and that only de-identified clinical information would be used in the evaluation. However, the NHSRC advised the team to proceed with consenting stating depressed patients are a vulnerable group and to request for a waiver should consenting interfere with the evaluation or delay care. This decision may bias the evaluation as the process of consenting patients and returning them to the clinic flow does take time and mildly disrupts care. It also places the evaluation team members in a position to help patients navigate initiating HIV treatment and depression screening, assistance new ART initiators will not receive at the end of this project. Implementation science is a relatively new discipline, particularly in low and middle income countries [49]. Understanding the ethical requirements of such evaluations as compared to other human subject research may require continued dialogue to ensure the objectives of implementation science can be achieved.

## Discussion

This activity has demonstrated how a depression screening program can successfully be integrated into HIV care within the public health system in Malawi. After seven months, between 88 and 93% of all newly diagnosed patients have been screened with the PHQ-2 and between 88 and 91% of those who score above 0 on the PHQ-2 have been screened with the entire PHQ-9. While limited research in the field of depression and HIV care integration has been conducted in sub-Saharan Africa, a few studies conducted in Tanzania, Zimbabwe, and Cameroon demonstrated that integration is possible [35–38]. As well, a clustered randomized control trial on integrating depression screening and treatment into HIV care in Uganda found that similar integration models (another algorithm-based model versus a model where ART clinicians individually decide how to further assess and treat depressed patients) could be successfully adopted and used to screen and treat depressed patients [40]. These findings provide evidence that with careful attention to implementation process HIV care providers can successfully screen individuals initiating HIV care for depression in Sub-Saharan Africa.

Previous prevalence estimates of depression in Malawi ranged from 9 to 30% [1–3, 50, 51]. While 25% of patients enrolled in our program evaluation reported symptoms of mild-to-severe depression, the evaluation revealed a relatively low (6%) prevalence of moderate-to-severe depression. This low prevalence may be due to our choice of screening tool, inconsistent administration and interpretation of the PHQ-9, or unaccounted for differences in how Malawians express and report symptoms of depression. Other studies have raised similar concerns [46, 52], suggesting further research on differences between depression screening tools and on cultural-constructs of depression may be warranted.

Key lessons learned from the implementation process show the importance of utilizing existing processes and infrastructure and focusing on iterative and collaborative learning. As well, efforts to address challenges around establishing a sense of program ownership among the HTC counselors and HIV providers, continuing to develop capacity to diagnose and manage depression, and ensuring the availability of appropriate medication provide insight into the technical and managerial support needed to prepare for, roll out, and sustain integrated models of mental health and HIV care. Similarly, the small study integrating depression treatment into HIV care in Tanzania using only two HIV providers also found that provider comfort prescribing anti-depressants and anti-depressant availability were challenging [3, 38]. However, the randomized control trial conducted in Uganda did stress that task-shifting could be successful with appropriate training, supervision and mentorship [40]. To our knowledge, no programmatic research evaluating programs integrating depression screening and treatment into HIV care in Sub-Saharan Africa has been published. Further implementation science research should be conducted in the region to better understand how to best integrate depression management into HIV care.

There are several limitations to this work. The results from this manuscript largely draw on the experiences of the implementers. While we believe it is important to share these experiences, we also recognize that the challenges we have noticed and highlighted here may be biased. As well, the prevalence of depression presented in this manuscript is estimated only from those individuals who consented to have their clinical data abstracted. Finally, this depression screening program was tailored for the Malawi health care context, specifically for public HIV clinics, and, as such, some findings may not be generalizable to other settings. However, we hope that sharing the experience of developing and implementing this program may serve as a guide for implementation of other integrated NCD and HIV programs in the region.

## Conclusions

This paper describes the many factors that must be taken into account during the implementation of a program integrating depression screening and treatment into HIV care in Malawi. A focus on integration and collaboration was key to successfully launching the program. However, fostering ownership, continuing to build capacity, mobilizing human resources and medication, and determining appropriate ethical standards for implementation science research in a clinical setting remained challenging. While this program focuses on integrating mental health care into HIV care, most of the lessons learned are not specific to HIV care, but would apply to integration of mental health into any non-specialist setting.

## Additional Files

Additional file 1: “Patient Health Questionnaire” The depression screening tool and the suicide risk assessment protocol in both English and Chichewa. (DOCX 113 kb)

Additional file 2: “Mental Health Mastercard” The clinical form used by providers to capture mental health screening and treatment data. (DOCX 113 kb)

Additional file 3: “Clinic Reference Guide” Posters used to remind providers how to administer and interpret the PHQ-9. (DOCX 92 kb)

## Abbreviations

ABCD: Algorithm-based Care for Depression
ART: Antiretroviral Treatment
DHMT: District Health Management Team
DHO: District Health Office
EMR: Electronic Medical Record
HIV: Human Immunodeficiency Virus
HSSP: Health Sector Strategic Plan
HTC: HIV Testing and Counseling
IRB: Institutional Review Board
MOH: Ministry of Health
NCDs: Non-communicable Diseases
NHSRC: National Health Science Research Committee
PEPFAR: United States President’s Emergency Plan for AIDS Relief
PHQ-2: Patient Health Questionnaire-2
PHQ-9: Patient Health Questionnaire-9
PST: Problem-solving Therapy
SOAR: Supporting Operational AIDS Research
UNAIDS: Joint United Nations Programme on HIV/AIDS
UNC: University of North Carolina
USAID: United States Agency for International Development
