# Supplementary material for: Integrating depression management into HIV primary care in central Malawi: the implementation of a pilot capacity building program
Source: BMC Health Serv Res. 2018 Jul 31;18:593. doi: 10.1186/s12913-018-3388-z (PMC6069990; doi:10.1186/s12913-018-3388-z)
Supplement: Supplementary file 1 — “Patient Health Questionnaire” The depression screening tool and the suicide risk assessment protocol in both English and Chichewa. (DOCX 113 kb) [file 12913_2018_3388_MOESM1_ESM.docx]

**Date: __________________**

**PATIENT PASSPORT LABEL**

**Patient Health Questionnaire**

***READ EACH QUESTION EXACTLY AS WRITTEN.***

***For every question, emphasize “In the past two weeks”***

***For every question, make sure the patient tells you the number of days they have experienced this symptom.***

| MARK ONE ANSWER FOR EACH QUESTION. | | | 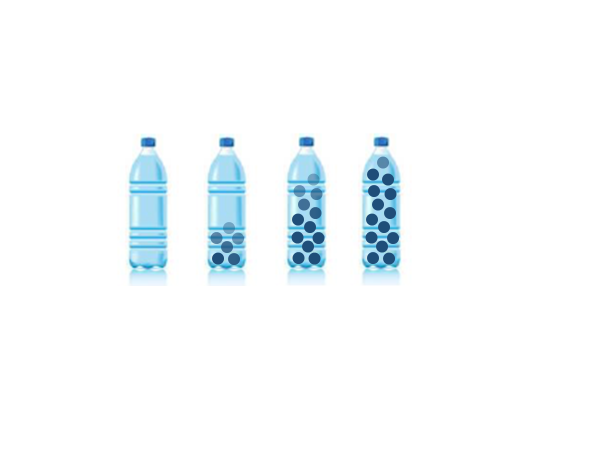 | 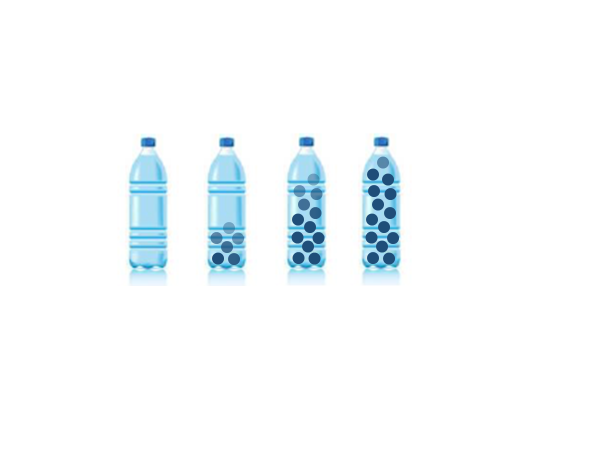 | 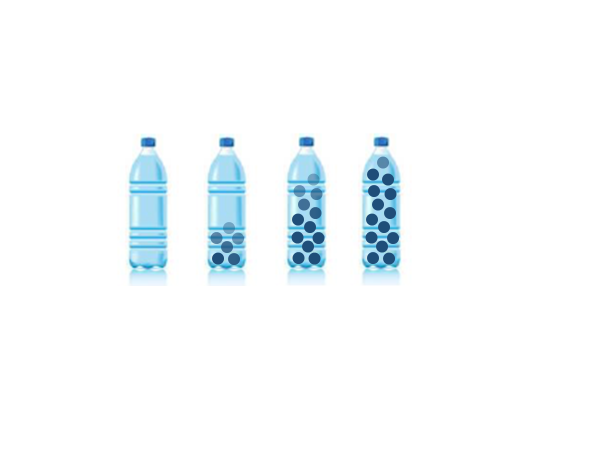 | 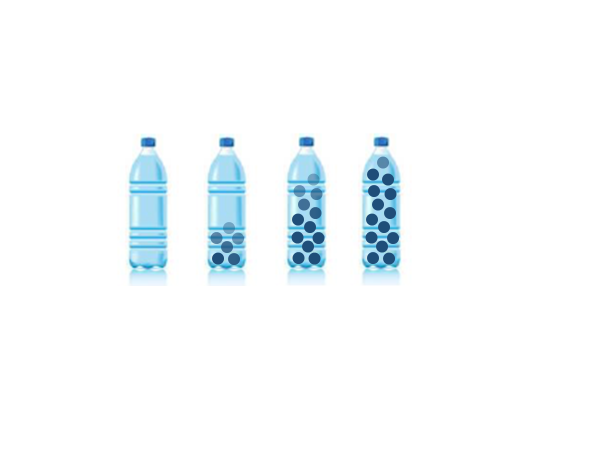 |
| --- | --- | --- | --- | --- | --- | --- |
| **PHQ-2** | 1. During the past two weeks, how many days have you been bothered by feeling down, depressed, or hopeless? | | 0 days  (0) | 1-7 days  (1) | 8-12 days  (2) | 13 or 14 days  (3) |
|  | 2. During the past two weeks, how many days have you been bothered by little interest or pleasure in doing things? | | 0 days  (0) | 1-7 days  (1) | 8-12 days  (2) | 13 or 14 days  (3) |
| **PHQ-2 Total Score (total of questions 1-2)** | | | | | |  |
| **If this is a screening and PHQ-2 score=0. STOP.**  **If this is a screening and PHQ-2 score>0, CONTINUE with questions 3-9.**  **If this is a follow-up for a patient with a previous PHQ-9 ≥5, CONTINUE with questions 3-9 regardless of PHQ-2 score.** | | | | | | |
| **PHQ-9** | 3. During the past two weeks, how many days have you been bothered by trouble falling or staying asleep, or sleeping too much? | | 0 days  (0) | 1-7 days  (1) | 8-12 days  (2) | 13 or 14 days  (3) |
|  | 4. During the past two weeks, how many days have you been bothered by feeling tired, fatigued or having little energy? | | 0 days  (0) | 1-7 days  (1) | 8-12 days  (2) | 13 or 14 days  (3) |
|  | 5. During the past two weeks, how many days have you been bothered by poor appetite or overeating? | | 0 days  (0) | 1-7 days  (1) | 8-12 days  (2) | 13 or 14 days  (3) |
|  | 6. During the past two weeks, how many days have you been bothered by feeling bad about yourself—or that you are a failure or have let yourself or your family down? | | 0 days  (0) | 1-7 days  (1) | 8-12 days  (2) | 13 or 14 days  (3) |
|  | 7. During the past two weeks, how many days have you been bothered by trouble concentrating on things, such as reading, talking with friends, finishing tasks, or attending a meeting? | | 0 days  (0) | 1-7 days  (1) | 8-12 days  (2) | 13 or 14 days  (3) |
|  | 8. During the past two weeks, how many days have you been bothered by moving or speaking so slowly that other people could have noticed? Or the opposite—being so fidgety or restless that you have been moving around a lot more than usual? | | 0 days  (0) | 1-7 days  (1) | 8-12 days  (2) | 13 or 14 days  (3) |
|  | 9. During the past two weeks, how many days have you been bothered by thoughts that you would be better off dead or of hurting yourself in some way? | | 0 days  (0) | 1-7 days  (1) | 8-12 days  (2) | 13 or 14 days  (3) |
|  | **IF ANSWER TO QUESTION 9 IS >0, COMPLETE SUICIDE RISK ASSESSMENT PROTOCOL (ON BACK) AND INDICATE ASSESSMENT RESULT:** | Suicide assessment result is:  **🞐 Passive 🞐 Active: Moderate/high risk**  **🞐 Active: Low risk 🞐 Active: Acute risk** | | | | |
|  | If suicide assessment risk is active-moderate to high or active-acute: Notes on suicide risk management plan: | | | | | |
| **PHQ-9 Total Score (total of questions 1-9)** | | | | | |  |

**CONTINUE TO BACK**

**Medication Side Effects**

***Instructions: Ask these questions only if the patient is taking mental health medications.***

| Please think about side effects you may be experiencing that you believe are due to the medication you are taking for your depression. | | | | | | | |
| --- | --- | --- | --- | --- | --- | --- | --- |
| **Freq.** | 1. Over the past week, how frequently (how often) have you experienced side effects from the medication you are taking for your depression? | 0  Not at all | 1  Less than half the time | | 2  About half the time | 3  More than half the time | 4  All the time |
| **Severi.** | 2. Over the past week, how intense have the side effects been from the medication you are taking for your depression? | 0  No side effects | 1  Mild | | 2  Moderate | 3  Severe | 4  Intolerable |
| **Interfer.** | 3. Over the past week, how much have the side effects you are experiencing interfered with your day-to-day functions? | 0  No inter-ference | 1  Mild inter-ference | | 2  Moderate interference | 3  Severe inter-ference | 4  Unable to function |
| **Type** | What side effects have you been experiencing? (Circle all that apply.) | **N** Nausea  **AG** Agitation  **AP** Abdominal pain  **AX** Anxiety  **BV** Blurred vision  **C** Constipation  **D** Diarrhoea | | **DM** Dry mouth  **DZ** Postural hypotension (‘dizziness when standing’)  **HP** Tachycardia/Arrhythmia (‘heart beating quickly’)  **R** Reflux | | **SE** Sedation  **SI** Sleep difficulties  **SX** Sexual problems  **U** Urinary retention  **V** Vomiting  **OT** Other:_________________ | |

**Suicide Risk Assessment Protocol (SRAP)**

**Complete this protocol with all patients who reveal suicidal thinking in their response to question 9 on PHQ-9 (score >0 on Q9 of PHQ-9).**

**I. Differentiation of passive from active suicidal thoughts**

**“*In the last two weeks, have you had any thoughts of hurting yourself in some way?”***

1 – not at all 2 - several days 3 - more than half the days 4 - nearly every day

**If “NOT AT ALL”: Very low risk (passive suicidal thoughts only). Skip to Section III.**

**OTHERWISE: Active suicidal thoughts. Continue with Section II.**

**II. Assessment of patients who demonstrate some evidence of *active* suicidal thinking.**

|  |  | **Yes** | **No** | **Comment** |
| --- | --- | --- | --- | --- |
| 1. | In the past month, have you made any plans or considered a method that you might use to harm yourself. *(*If yes, ask “*Please be specific about these plans or methods you have considered.”)* |  |  |  |
| 2. | Have you ever attempted to harm yourself? (If yes, ask, “*When was this? What happened*?”) |  |  |  |
| 3. | There’s a big difference between having a thought and acting on a thought. Do you think you might actually make an attempt to hurt yourself in the near future? (If yes, ask, “*Can you be specific about how you might do this*?”) |  |  |  |
| 4. | In the past month have you told anyone that you were going to commit suicide, or threatened that you might do it? (If yes, ask, “*Who have you told and what have you said to them?*”) |  |  |  |
| 5. | Do you think there is any risk that you might hurt yourself before you see your doctor the next time? (If yes, ask, “*What do you think you might do?”*) |  |  |  |

**If “YES” to Question 5: Acute (High) Suicide Risk.**

**Otherwise, if “YES” to any of Questions 1-4: Moderate to High Suicide Risk**

**If “NO” to ALL of Questions 1-5: Low Suicide Risk**

**III. Summary of risk assessment. Check one.**

__ Passive (very low) __ Low __ Moderate to high __ Acute

Assessment completed by: ____________________________________________________________

Name Signature

**Date: __________________**

**PATIENT PASSPORT LABEL**

**Patient Health Questionnaire**

***READ EACH QUESTION EXACTLY AS WRITTEN.***

***For every question, emphasize “In the past two weeks”***

***For every question, make sure the patient tells you the number of days they have experienced this symptom.***

|  | | 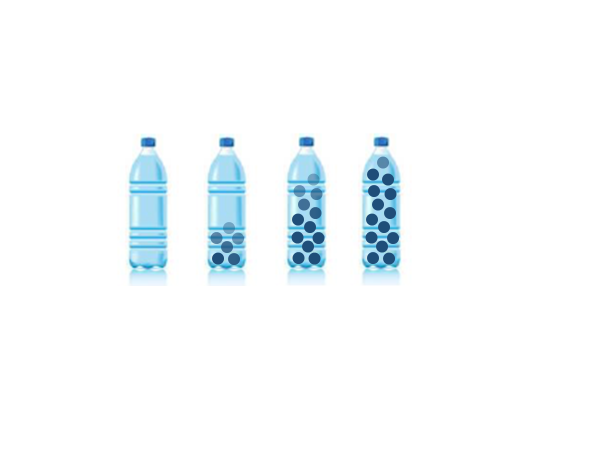 | | 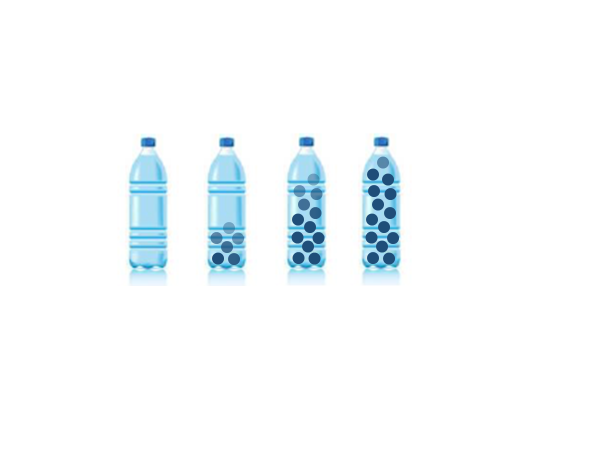 | 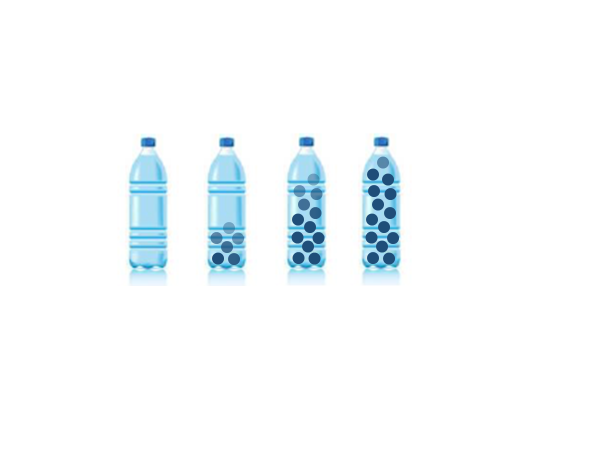 | 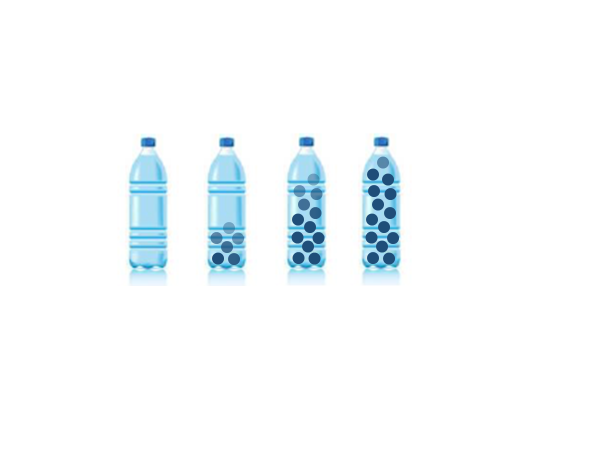 |
| --- | --- | --- | --- | --- | --- | --- |
| **PHQ-2** | 1. **Mumasabata awiri apitawa**, ndikangati munavutika ndi kukhumudwa kapena kupanda chiyembekezo? | Palibe ndi tsiku limodzi lomwe  (0) | | Masiku 1-7  (1) | Masiku  8-12  (2) | Masiku 13 or 14  (3) |
|  | 2**. Mumasabata awiri apitawa,** ndikangati munavutika kukhala ndi chidwi chochepa kapena kusasangalatsidwa mkuchita zinthu (osalimba mtima kapena nkhawa )? | Palibe ndi tsiku limodzi lomwe  (0) | | Masiku 1-7  (1) | Masiku  8-12  (2) | Masiku 13 or 14  (3) |
| **PHQ-2 Total Score (total of questions 1-2)** | | | | | |  |
| **If this is a screening and PHQ-2 score=0. STOP.**  **If this is a screening and PHQ-2 score>0, CONTINUE with questions 3-9.**  **If this is a follow-up for a patient with a previous PHQ-9 ≥5, CONTINUE with questions 3-9 regardless of PHQ-2 score.** | | | | | | |
| **PHQ-9** | 3. **Mumasabata awiri apitawa**, ndikangati mwakhala mukuvutika kupeza tulo kapena kugona moposa nthawi zonse? | Palibe ndi tsiku limodzi lomwe  (0) | | Masiku 1-7  (1) | Masiku  8-12  (2) | Masiku 13 or 14  (3) |
|  | 4. **Mumasabata awiri apitawa**, ndikangati mwakhala mu kufooka, kukhala ndi ulesi kapena kuchepekedwa mphamvu? | Palibe ndi tsiku limodzi lomwe  (0) | | Masiku 1-7  (1) | Masiku  8-12  (2) | Masiku 13 or 14  (3) |
|  | 5. **Mumasabata awiri apitawa**, ndikangati mwakhala mukusowa chilakolako cha chakudya kapena kudya kwambiri? | Palibe ndi tsiku limodzi lomwe  (0) | | Masiku 1-7  (1) | Masiku  8-12  (2) | Masiku 13 or 14  (3) |
|  | 6. **Mumasabata awiri apitawa**, ndikangati munavutika ndi *kuzimvera chisoni -*kapena kuziona olephera/*osafunikila/onyozeka kapena kuti mwapangitsa manyazi a banja lanu* | Palibe ndi tsiku limodzi lomwe  (0) | | Masiku 1-7  (1) | Masiku  8-12  (2) | Masiku 13 or 14  (3) |
|  | 7. **Mumasabata awiri apitawa**, ndikangati munavutika ndikulingalira kapena kukhazikika pa chinthu monga kuwerenga, kucheza ndi anzanu, kuchita nawo zokambirana pamsonkhano | Palibe ndi tsiku limodzi lomwe  (0) | | Masiku 1-7  (1) | Masiku  8-12  (2) | Masiku 13 or 14  (3) |
|  | 8. **Mumasabata awiri apitawa**, ndikangati mwakhala mukuyenda kapena kuyankhula mochedwa zoti mpakana anthu ena kudabwa kapena kusakhazikika nkumangoyenda yenda moposera muyeso? | Palibe ndi tsiku limodzi lomwe  (0) | | Masiku 1-7  (1) | Masiku  8-12  (2) | Masiku 13 or 14  (3) |
|  | 9. **Mumasabata awiri apitawa**, ndikangati mwakhala ndi malingaliro akuti kuli bwino kungofa kapena kuzivulaza nokha mwa njira ina yake? | Palibe ndi tsiku limodzi lomwe  (0) | | Masiku 1-7  (1) | Masiku  8-12  (2) | Masiku 13 or 14  (3) |
|  | **IF ANSWER TO QUESTION 9 IS >0, COMPLETE SUICIDE RISK ASSESSMENT PROTOCOL (ON BACK) AND INDICATE ASSESSMENT RESULT:** | | Suicide assessment result is:  **🞐 Passive**  **🞐 Active – Low risk**  **🞐 Active – Moderate to high risk**  **🞐 Active – Acute risk** | | | |
|  | If suicide assessment risk is active-moderate to high or active-acute: Notes on suicide risk management plan: | | | | | |
| **PHQ-9 Total Score (total of questions 1-9)** | | | | | |  |

**CONTINUE TO BACK**

**Medication Side Effects**

***Instructions: Ask these questions only if the patient is taking mental health medications.***

| Chonde ganizirani zovuta ndi zosowetsa mtendere zomwe mwakhala mukumva zomwe mukukhulupirira kuti ndi chifukwa cha mankhwala omwe mukumwa a matenda okhumudwa. | | | | | | | | |
| --- | --- | --- | --- | --- | --- | --- | --- | --- |
| **Freq.** | 1. Pa mulungu wathawu, ndi kangati mwapezeka ndi zovuta ndi zosowetsa mtendere kuchokera ku mankhwala amene mukumwa a matenda anu okhumudwa? | 0  Palibe | 1  Kuchepera theka la nthawi | | 2  Pafupifupi theka la nthawii | 3  Kupyolera theka la nthawi | | 4  Nthawi zonse |
| **Severity** | 2. Pa mulungu wathawu, kodi ndi kukula kotani kwa zovuta ndi zosowetsa mtendere kwakhala kukuchitika kuchokera ku mankhwala anu amene mukumwa a kukhumudwa? | 0  Palibe zosagwirizana ndi mankhwala | 1  Pang’ono zedi | | 2  Pang’ono | 3  Kwambiri | | 4  Kosapilirika |
| **Interfer.** | 3. Pa mulungu wathawu, kodi zovuta ndi zosowetsa mtendere zomwe mukukumana nazo zasokoneza mochuluka bwanji kagwiridwe kanu ka ntchito za tsiku ndi tsiku? | 0  Palibe kusokoneza | 1  Zasokoneza Pang’ono zedi | | 2  Zasokoneza Pang’ono | 3  Zasokoneza Kwambiri | | 4  Osagwira nako ntchito |
| **Type** | Ndi zovuta ndi zosowetsa mtendere ziti zimene mwakhala mukumva?  (Circle all that apply.) | **N** Nausea  **AG** Agitation  **AP** Abdominal pain  **AX** Anxiety  **BV** Blurred vision  **C** Constipation  **D** Diarrhoea | | **DM** Dry mouth  **DZ** Postural hypotension (‘dizziness when standing’)  **HP** Tachycardia/Arrhythmia (‘heart beating quickly’)  **R** Reflux | | | **SE** Sedation  **SI** Sleep difficulties  **SX** Sexual problems  **U** Urinary retention  **V** Vomiting  **OT** Other:_________________ | |

**Suicide Risk Assessment Protocol (SRAP)**

**Complete this protocol with all patients who reveal suicidal thinking in their response to question 9 on PHQ-9 (score >0 on Q9 of PHQ-9).**

**I. Kusiyanitsa kwa mulingo wamaganizo ofuna kuzipha**

**“*Pa masabata awiri apitawo, munakhalapo ndi maganizo ofuna kuzivulaza nokha munjira ina iliyonse?”***

1 – Ayi 2 – Eya, kwa masiku angapo 3 – Eya, kuposera theka la masiku onse 4 –Eya, pafupifupi tsiku lirilonse

**Ngati “AYI”: Ali pa chiwopsyezo chochepa kwambiri. Pitani ku gawo III.**

**Koma ngati “EYA”: Ali pachiwopsyezo. Pitani ku gawo II.**

**II. Muyeso wothandizira kuona mulingo wachiopsyezo chofuna kuzipha yekha.**

|  |  | **Eya** | **Ayi** | **Ndemanga** |
| --- | --- | --- | --- | --- |
| 1. | Mwezi wapitawu, munapangako ganizo lofuna kuzipha kapena dongosolo lofuna kuziphera. *(*ngati eya, “*longosolani mwasatanesatane njira imene munakonza yofuna kuziphera.”)* |  |  |  |
| 2. | Munayamba mwayeserapo kufuna kuzipha nokha mbuyomu? (ngati eya, “*linali liti? Chinachitika ndichiyani*?”) |  |  |  |
| 3. | Pali kusiyana pakati pakukhala ndi malingaliro ofuna kuzipha ndi mchitidwe ofuna kuzipha. Inuyo mukuona ngati mungafune kuzivulaza mwadala posachedwapa? (ngati eya, “*longosolani mwasatanesatane kuti muzapanga bwanji*?”) |  |  |  |
| 4. | Mwezi wapitawu, munamuwuzapo wina aliyense kuti mukufuna mutazipha kapena kuwopsyeza kuti muzipha? (ngati eya, “*munawuzapo ndani ndipo anakuwuzani chani?*”) |  |  |  |
| 5. | Kodi mukuwona ngati muli pachiwopsyezo choti mutha kuzivulaza nokha pamasiku amene mukuyembekezera kuzaonana ndi adotolo? (ngati eya, “*mukuona ngati mungapange chani?”*) |  |  |  |

**Ngati “EYA”, kufunso namba 5: Chiwopsyezo chachikulu kwambiri.**

**Koma, ngati “EYA’’, kufunso lina liliyonse kwamafunso 1-4: Chiwopsyezo chachikulu**

**Ngati “AYI’’, kumafunso 1-5: Chiwopsyezo chochepa**

**III. Mulingo wachiwopsyezo chofuna kuzipha: Chongani malo amodzi.**

__ chochepa kwambiri __ chochepa __ chachikulu __ chachikulu kwambiri

Assessment completed by:

______________________________________________________________________________

Name Signature
