# Supplementary figures and images for: Integrating depression management into HIV primary care in central Malawi: the implementation of a pilot capacity building program
Source: BMC Health Serv Res. 2018 Jul 31;18:593. doi: 10.1186/s12913-018-3388-z (PMC6069990; doi:10.1186/s12913-018-3388-z)

**Mental Health Mastercard**


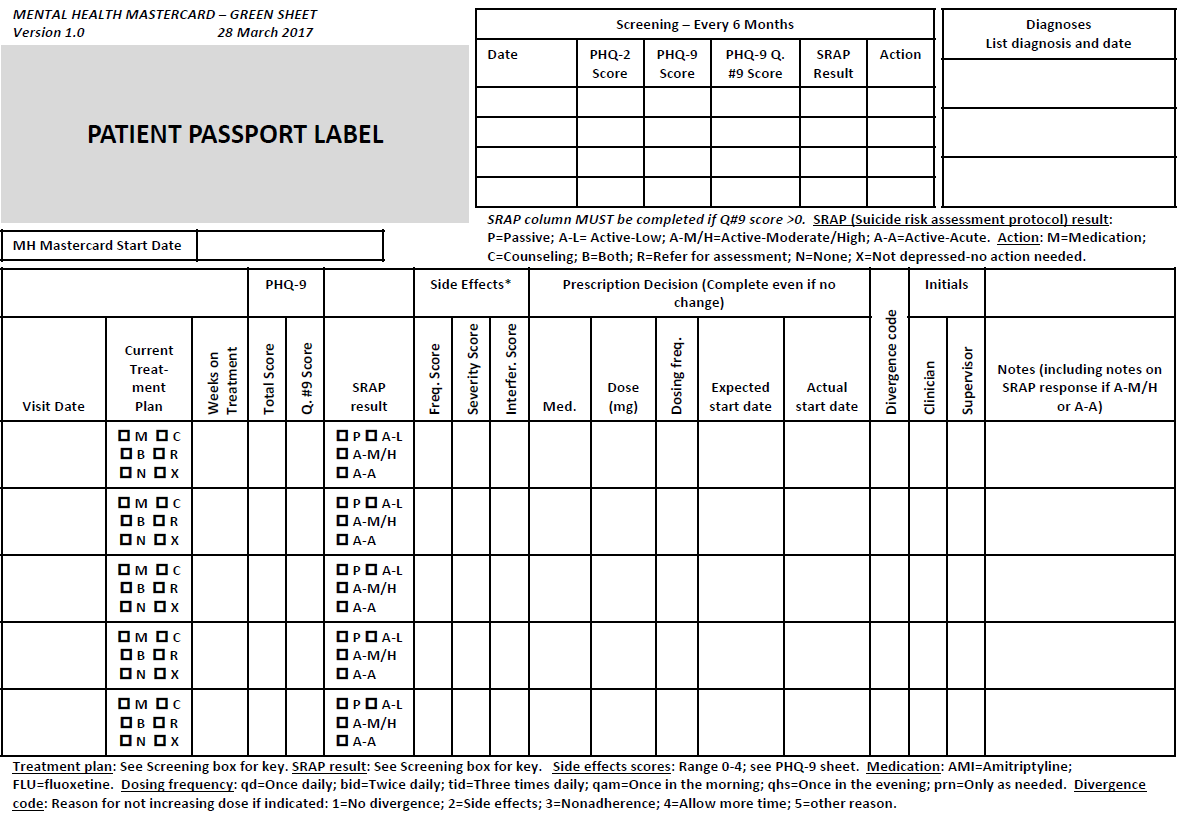

Supplement: Supplementary file 2 — “Mental Health Mastercard” The clinical form used by providers to capture mental health screening and treatment data. (DOCX 113 kb) [file 12913_2018_3388_MOESM2_ESM.docx]

**Clinic Reference Guide**


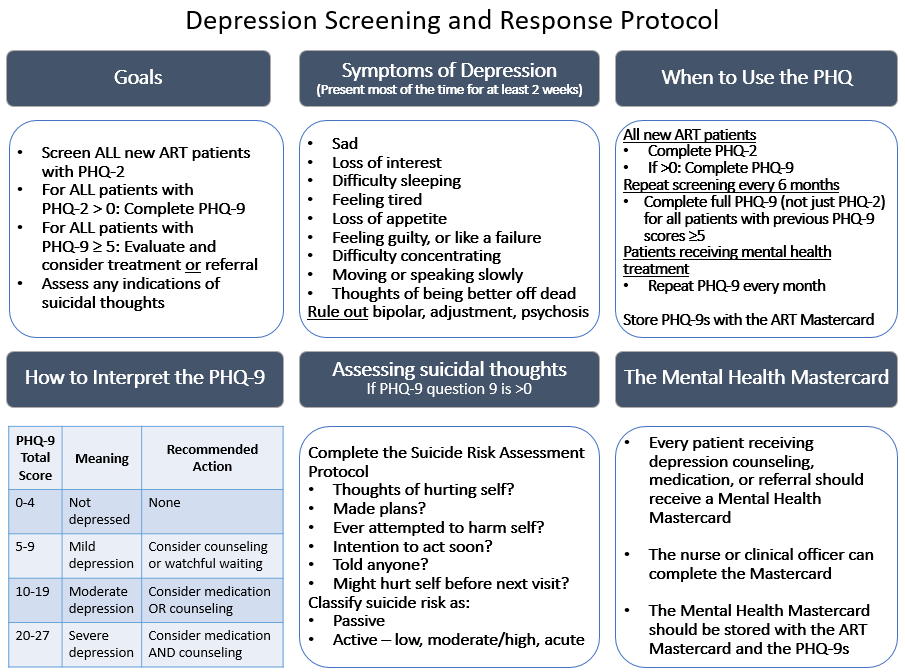

Supplement: Supplementary file 3 — “Clinic Reference Guide” Posters used to remind providers how to administer and interpret the PHQ-9. (DOCX 92 kb) [file 12913_2018_3388_MOESM3_ESM.docx]
